# Lack of Influence of Thyroid Hormone on Bone Mineral Density and Body Composition in Healthy Euthyroid Women

**DOI:** 10.3389/fendo.2019.00890

**Published:** 2020-01-10

**Authors:** Denise Engelbrecht Zantut-Wittmann, Alessandra Quintino-Moro, Priscilla Nazaré Silva dos Santos, Vaneska Melhado-Kimura, Luís Bahamondes, Arlete Fernandes

**Affiliations:** ^1^Division of Endocrinology, Department of Internal Medicine, São Paulo, Brazil; ^2^Family Planning Clinic of Department of Obstetrics and Gynecology, School of Medical Sciences, University of Campinas, São Paulo, Brazil

**Keywords:** thyroid hormone, bone mineral density, body composition, healthy women, euthyroidism

## Abstract

**Objective:** The objective of this study was to evaluate whether evolution of bone mineral density (BMD) is associated with the thyroid hormone profile in a cohort of euthyroid women with no other known diseases within 1 year.

**Methods:** This was a prospective cohort study conducted at the University of Campinas, Brazil. We used a database with 52 women aged 20–39 who were followed for 1 year in a family planning outpatient clinic. The inclusion criteria were body mass index (BMI) <30 kg/m^2^, no known diseases/medication use, fasting glucose <100 mg/dl, and 2 h glucose after a 75 g oral glucose load <140 mg/dl. The women were divided into groups of normal weight (*n* = 30) and overweight (*n* = 22). The main outcomes were BMD measured by dual-energy x-ray absorptiometry (DXA) and thyroid hormone profile (thyrotropin TSH, free triiodothyronine FT3, free thyroxine FT4, and T3/T4 ratio); other variables were body composition (DXA), calcium metabolism markers, and life habits. We used a repeated measures analysis of variance (ANOVA) and multiple regression analyses to evaluate associations.

**Results:** At the baseline data collection, overweight women showed a higher T3/T4 ratio, leptin, calcium, BMD in the lumbar spine and total femur, total mass, mass, and percentage of fat mass than normal weight women. At 12 months, both groups had increased FT4, calcium, ALP, femoral neck BMD, and total mass by time effect. The normal weight group presented a decrease of vitamin D when compared to the baseline. Increased BMD of the femoral neck was associated with moderate coffee intake, and as such, there were no associations found between this increase and the thyroid hormone profile. Leptin and ALP were associated with total mass variation, while leptin and PTH were associated with fat mass variation. The normal BMI was inversely associated with the variation of total mass, mass, and percentage of fat mass, and engaging in regular physical activity was inversely associated with fat mass variation.

**Conclusions:** In this sample of euthyroid healthy women who were both normal weight and overweight, the thyroid hormone profile was not associated with variations in bone mineral density and body composition after a 1 year follow-up.

## Introduction

Thyroid hormones have an effect on the entire postnatal period of formation, growth, and development of the skeleton. In adults, thyroid hormones play a role in the maintenance of bone mass quality. The effect of T3 on the hypothalamic–pituitary axis is exerted through beta-thyroid hormone receptors (TRβ). Bone and cartilage express α and β TR receptors. However, the main TR isoform that regulates the actions of thyroid hormones in the osteoblasts and chondrocytes is TRα, resulting in the agonist action for bone formation, growth, and mineralization (1).

A meta-analysis was conducted involving 12 prospective studies in which 56,835 adult subjects were involved. Five percent of the subjects had hip fractures. The results showed that euthyroid individuals presented a 25% risk for hip fracture when their TSH concentrations were at the lower limit of the reference range, and when their FT4 were at the upper limit (2). Several other studies have evaluated adult euthyroid individuals with respect to thyroid hormones and their interaction with bone mass behavior. However, due to the different designs and characteristics of the populations studied, there is currently no consensus on whether a variation in serum concentration of thyroid hormones within the euthyroid serum range affects bone metabolism and consequently bone mineral density (BMD) and fracture risk (3–7).

Adult thyroid dysfunctions appear to impact bone mass negatively, as evidenced by intense bone reabsorption leading to osteoporosis and an increased risk of fracture associated with thyrotoxicosis (8, 9). On the other hand, there is no evidence to suggest that these changes are associated with hypothyroidism (10, 11). Still, there are numerous factors that can influence the quality of the bone, in addition to genetic factors. Age, body weight and composition, life habits, and geographical location can have different effects on the bone health of an individual (12–14).

Thyroid dysfunctions can cause variations in body composition and weight. However, euthyroid individuals also present wide variations in these measures. On the other hand, studies on body composition and weight variation in relation to thyroid hormone levels within reference values have shown conflicting results (15–17).

In order to study a more homogeneous population, we chose to evaluate the evolution of BMD and body composition in relation to the thyroid hormone profile in adult euthyroid women with no known diseases.

## Materials and Methods

### Study Design

We conducted a prospective cohort study at the Department of Obstetrics and Gynecology and at the Endocrinology Unit of the Department of Internal Medicine, School of Medical Sciences, University of Campinas (UNICAMP) in São Paulo, Brazil. We used a previous research database of women using two types of contraceptive methods—either depot medroxyprogesterone acetate (DMPA) or a copper intrauterine device (IUD)—and whose main objective was to evaluate the insulin resistance (18). The university's internal review board approved the principal study protocol, and all volunteers signed an informed consent form prior to enrolment.

The main study included women aged 18–39 years, with a body mass index (BMI) <30 kg/m^2^, with no known history of disease and no knowledge of a first-degree family history of chronic disease. In this study, we analyzed a database of 52 women aged 20–39 years at baseline and again at 12 months to assess their thyroid function, BMD, and body composition. We adjusted all statistical analyses by using the DMPA contraceptive method.

The women were recruited via Basic Health Units. Inclusion criteria for this sample included a history of prior DMPA use, along with normal results on an oral glucose tolerance test (fasting glucose levels <100 mg/dl and 2 h glucose levels <140 mg/dl). The exclusion criteria entailed whether the women were currently breastfeeding or had personal or first-degree family history of type 1 or 2 diabetes mellitus, any chronic disease, hirsutism, or polycystic ovary syndrome. Participants were also excluded if they regularly used any drug that may be associated with weight gain and/or the development of insulin resistance, or if they had a chronic history of corticosteroid, antipsychotic, statin, or thiazide use.

### Procedures

We studied the database of all 52 women at baseline and after 12 months (18). The variables used included the following: initial measures of BMI (kg/m^2^), thyroid function profile including serum markers of calcium metabolism, and measures of BMD and body composition (BC) assessed by dual-energy x-ray dual absorptiometry (DXA) using the Lunar DPX (GE Healthcare, Lunar Corporation, Madison, WI, USA) densitometer. Data from the following measurements were collected and analyzed: BMD measurements of the total lumbar spine (L1–L4), femoral neck and total femur, along with BC measurements of the total mass, fat mass and lean mass, including the percentage of fat mass.

This sample of women used a hormonal contraceptive method—either DMPA or a non-hormonal copper IUD. All women were followed for 12 months. They returned to the Family Planning Service after 3 months, where they received an orientation on healthy eating habits, mainly in relation to maintaining bone health, and regular physical exercise. Initial BMI was calculated using the formula weight (kg)/height (m) squared and the patients were categorized either as having a normal weight (18.5 ≤ BMI < 25 kg/m^2^) or as being overweight (25 ≤ BMI < 30 kg/m^2^) (19).

Participants were asked to record the food that they ingested, including portion sizes, at both the baseline and 12 month study. The data were reviewed by the same nutrition professional (20). The variables used were the daily intake of calcium (categorized as adequate: ≥1 g/day, inadequate: <1 g/day) and coffee consumption (light consumption: ≤200 mg caffeine/day or moderate consumption: >200 mg caffeine/day) with the quantities calculated by a software program (DietWin Professional® version 5.1, Porto Alegre, Brazil). The women also answered a questionnaire about daily physical activity habits, placing themselves in the category of either active (performing aerobic exercise for ≥150 min/week) or inactive (performing <150 min/week, in the last 3 months) (14), current smoker (≥100 cigarettes in a lifetime)/no, former smoker (<100 cigarettes in a lifetime), or never smoked (21), and time and regularity of exposure to the sun, categorized by weekly sun exposure. Sun exposure of at least 20% of body surface was self-reported by the participants and categorized as adequate (≥20 min/week or ≥3×/week) or inadequate exposure (<20 min/week or <3×/week) (13).

The technologies used to measure thyroid hormone levels in the blood samples given were electrochemiluminescence (Roche Hitachi-Elecsys Cobas, USA) to measure TSH (reference values [RV] 0.45–4.5 IU/L), free thyroxine (FT4, RV 0.9–1.8 ng/dl), and free triiodothyronine (FT3, RV 0.25–0.44 ng/dl). We calculated the FT3/FT4 ratio by simply dividing the values of the two hormone levels for surrogate measures of deiodinase activity. Levels of the adipokines leptin and adiponectin were determined using the Human Leptin “Dual Range” ELISA (sensitivity 0.2 ng/ml) and Human Adiponectin ELISA (sensitivity 0.2 ng/ml) assays, respectively, (Merck Millipore, Darmstadt, Germany). The quantitative determination of intact parathyroid hormone (PTH, RV 15–65 pg/ml) in serum was evaluated using electrochemiluminescence assays (PTH STAT Elecsys Cobas, Roche, Indianapolis, USA); the 25 hydroxyvitamin D [25(OH)D, RV ≥20 ng/ml] assay was performed by radioimmunoassay (kit sensitivity 1.5 ng/ml, Genesis Laboratory, São Paulo, Brazil), serum calcium was measured by the complex colorimetric method of Ca++ and Arsenazo III (RV 8.8–10.6 mg/dl), albumin was measured by bromocresol green colorimetric assay (RV 3,5–5.2 g/dl), alkaline phosphatase (ALP) was measured by kinetic colorimetric method (RV 30–120 U/L), and phosphorus (P) was measured by the phosphomolibidate method with an ultraviolet reading (RV 2.5–4.5 mg/dl).

### Statistical Analysis

We used Pearson's chi-square test or Fisher's exact test, when necessary, and the Mann–Whitney test to compare the categorical and numerical variables, respectively. We used the repeated-measures analysis of variance (ANOVA) to compare the groups across time points, with the response variables being transformed into ranks. All analyses were adjusted for the use of DMPA contraceptive. At the end, we performed the multiple linear regression analysis to evaluate the variables associated with BMD variation and with BC variation at 12 months in relation to baseline measurements. We adopted the significance level of 5%.

## Results

We evaluated 52 euthyroid non-obese women 20–39 years old, with mean BMI 24.19 ± 3.22 kg/m^2^ (median 23.93, 18.13–29.40). The patients were selected in two groups according to BMI: normal (BMI < 25 kg/m^2^, *n* = 30) or overweight (25 ≤ BMI < 30 kg/m^2^, *n* = 22). In this sample, we did not observe differences in the two time periods studied with relation to smoking habits, sun exposure, regular physical exercise, and calcium or coffee intake (data not shown).

### Comparative Analysis at Baseline Moment

As for the thyroid hormone profile, analyses of FT4, FT3, and TSH concentrations did not evidence differences between the groups. However, the T3/T4 ratio was significantly higher in overweight women than in normal weight women (*p* = 0.0372). Leptin concentration was significantly higher in overweight women than in normal women (0.0390).

Relating to calcium metabolism and vitamin D (calcium, phosphorus, paratormone, 25OHvitamin D, alcalin phosphatase), only the concentration of serum calcium was higher in overweight women than in normal women (0.0054) (Table 1).

**Table 1:** Comparison of serum concentration of thyroid hormones and biochemical markers between baseline and 12 months in women of normal weight and overweight.

	**Baseline**		**After 12 months**		**ANOVA^¥^**		
**Variables^@^**	**<25 kg/m^**2**^** ***n* = 30**	**≥25 kg/m^**2**^** ***n* = 22**	**<25 kg/m^**2**^** ***n* = 30**	**≥25 kg/m^**2**^** ***n* = 22**	**Comparison between groups, *p*-value**	**Time comparison, *p*-value**	**Time–group interaction, *p*-value**
TSH, IU/L, mean (SD)	1.80 (1.13)	1.86 (1.24)	1.65 (0.81)	1.69 (1.02)	0.8169^a^	0.6619	0.8257
Median	1.40	1.37	1.44	1.46	0.8274^b^		
Free T3, ng/dl, mean (SD)	0.32 (0.03)	0.33 (0.07)	0.33 (0.03)	0.32 (0.04)	0.7230^a^	0.1464	0.1590
Median	0.32	0.32	0.33	0.32	0.8569^b^		
Free T4, ng/dl, mean (SD)	1.20 (0.12)	1.18 (0.19)	1.28 (0.13)	1.25 (0.10)	0.2393^a^	<0.0001	0.9746
Median	1.19	1.15	1.29	1.25	0.2930^b^		
T3/T4 ratio, mean (SD)	0.26 (0.03)	0.28 (0.04)	0.26 (0.03)	0.26 (0.03)	0.0372^a^		
Median	0.27	0.29	0.26	0.26	0.4149^d^		
Leptin, ng/ml, mean (SD)	3.54 (0.83)^*^	4.02 (0.90)	3.47 (0.99)^*^	3.98 (1.01)	0.0390^a^		
Median	3.63	3.90	3.71	4.17	0.1889^d^		
Adiponectin, ng/ml, mean (SD)	7.25 (4.58)^*^	6.78 (2.77)	6.59 (4.18)^*^	7.28 (4.13)	0.8417^a^		
Median	5.98	5.75	5.01	5.55	0.5907^b^	0.2831	0.7649
PTH, pg/ml, mean (SD)	36.63 (14.28)	34.80 (14.35)	35.91 (10.87)	38.25 (12.06)	0.4582^a^	0.3547	0.1165
Median	35.95	30.79	35.67	37.38	0.9147^b^		
25(OH)D, ng/ml, mean (SD)	29.04 (10.04)	27.51 (9.57)	25.04 (7.19)	26.89 (6.35)	0.3974^a^	0.3922	0.0423^c^
Median	26.68	23.89	25.24	26.94	0.8681^b^		
Calcium, mg/dl, mean (SD)	8.58 (0.65)	8.80 (0.58)	9.07 (0.42)	9.10 (0.3)	0.0054^a^	<0.0001	0.1949
Median	8.70	9.00	9.00	9.1	0.1092^b^		
P, mg/dl, mean (SD)	3.40 (0.40)	3.37 (0.46)	3.62 (0.40)	3.50 (0.34)	0.2853		
Median	3.40	3.30	3.65	3.45			
ALP, U/L, mean (SD)	51.47 (16.97)	50.32 (15.98)	62.97 (15.51)	59.23 (14.67)	0.8529^a^	<0.0001	0.5008
Median	49.00	48.50	62.50	56.50	0.5542^b^		
Albumin, g/dl, mean (SD)	4.21 (0.45)	4.24 (0.48)	4.46 (0.33)	4.40 (0.27)	0.6892^a^	0.0015	0.3295
Median	4.25	4.30	4.40	4.40	0.9789^b^		

*PTH, intact parathyroid hormone; P, phosphorus; ALP, alkaline phosphatase; SD, standard deviation*.

@ *Variables transformed into ranks for testing due to lack of normal distribution*.

* *Missing = 1 (n = 29)*.

¥ *ANOVA for repeated measures, adjusted for the use of depot medroxyprogesterone acetate during follow-up. Differences between groups at baseline^a^ and at 12 months^b^. ^c^12 months < baseline for BMI <25, p = 0.0132. ^d^Differences between groups with adjustment for initial measurement (ANOVA)*.

As for BMD parameters, L1–L4 (*p* = 0.0177), and total femur (*p* = 0.0115) were significantly higher in overweight women than in normal women and not femur neck.

Regarding body composition, total mass (*p* < 0.0001), fat mass (*p* < 0.0001), and % of fat mass (*p* < 0.0001) were significantly higher in overweight women than in normal women and not lean mass (Table 2).

**Table 2:** Comparison of bone mineral density (BMD) and body composition between baseline and 12 months in women of normal weight and overweight.

	**Baseline**		**After 12 months**		**ANOVA^¥^**		
**Variables^@^**	**<25 kg/m^**2**^** ***n* = 30**	**≥25 kg/m^**2**^** ***n* = 22**	**<25 kg/m^**2**^** ***n* = 30**	**≥25 kg/m^**2**^** ***n* = 22**	**Comparison between groups, *p*-value**	**Time comparison, *p*-value**	**Time–group interaction, *p*-value**
BMD L1–L4, g/cm^2^, mean (SD)	1.15 (0.10)	1.23 (0.11)	1.14 (0.09)	1.21 (0.10)	0.0177^a^		
Median	1.16	1.21	1.14	1.23	0.6412^c^		
BMD fêmur neck, g/cm^2^, mean (SD)	0.98 (0.10)	1.02 (0.12)	0.96 (0.11)	1.01 (0.12)	0.3083^a^	0.0394	0.6039
Median	1.01	1.03	0.97	1.03	0.2072^b^		
BMD total femur, g/cm^2^, mean (SD)	0.94 (0.13)	1.03 (0.11)	0.96 (0.11)	1.02 (0.10)	0.0115^a^		
Median	0.96	1.04	0.96	1.03	0.2965^c^		
**Body composition**							
Total mass, kg, mean (SD)	55.24 (5.66)	67.27 (5.65)	57.00 (6.96)	67.46 (6.79)	< .0001^a^		0.0196
Median	55.60	67.95	55.70	67.15	0.0126^c^		
Fat mass, kg, mean (SD)	34.28 (6.08)	45.00 (4.80)	36.85 (5.46)	44.66 (5.25)	< .0001^a^		
Median	35.10	45.30	37.15	44.20	0.9918^c^		
% of Fat mass, mean (SD)	18.23 (4.14)	29.13 (4.46)	20.33 (4.72)	29.15 (5.63)	< .0001^a^		
Median	18.68	28.26	20.20	30.56	0.1680^c^		
Lean mass, kg, mean (SD)	34.64 (3.97)	35.44 (3.93)	34.28 (3.47)	35.66 (3.22)	0.7863^a^		
Median	34.88	34.28	34.37	34.76	0.0196^d^		

*BMD, bone mineral density; SD, standard deviation*.

@ *Variables transformed into ranks for testing due to lack of normal distribution. Differences between groups at baseline^a^ and at 12 months^b^*.

¥ *ANOVA for repeated measures, adjusted for the use of depot medroxyprogesterone acetate during follow-up. ^c^Differences between groups with adjustment for initial measurement (ANOVA). ^d^It was not possible to detect differences after independent evaluation of the time and group factor*.

### Comparative Analysis at 12 Months

**Figure 1:**
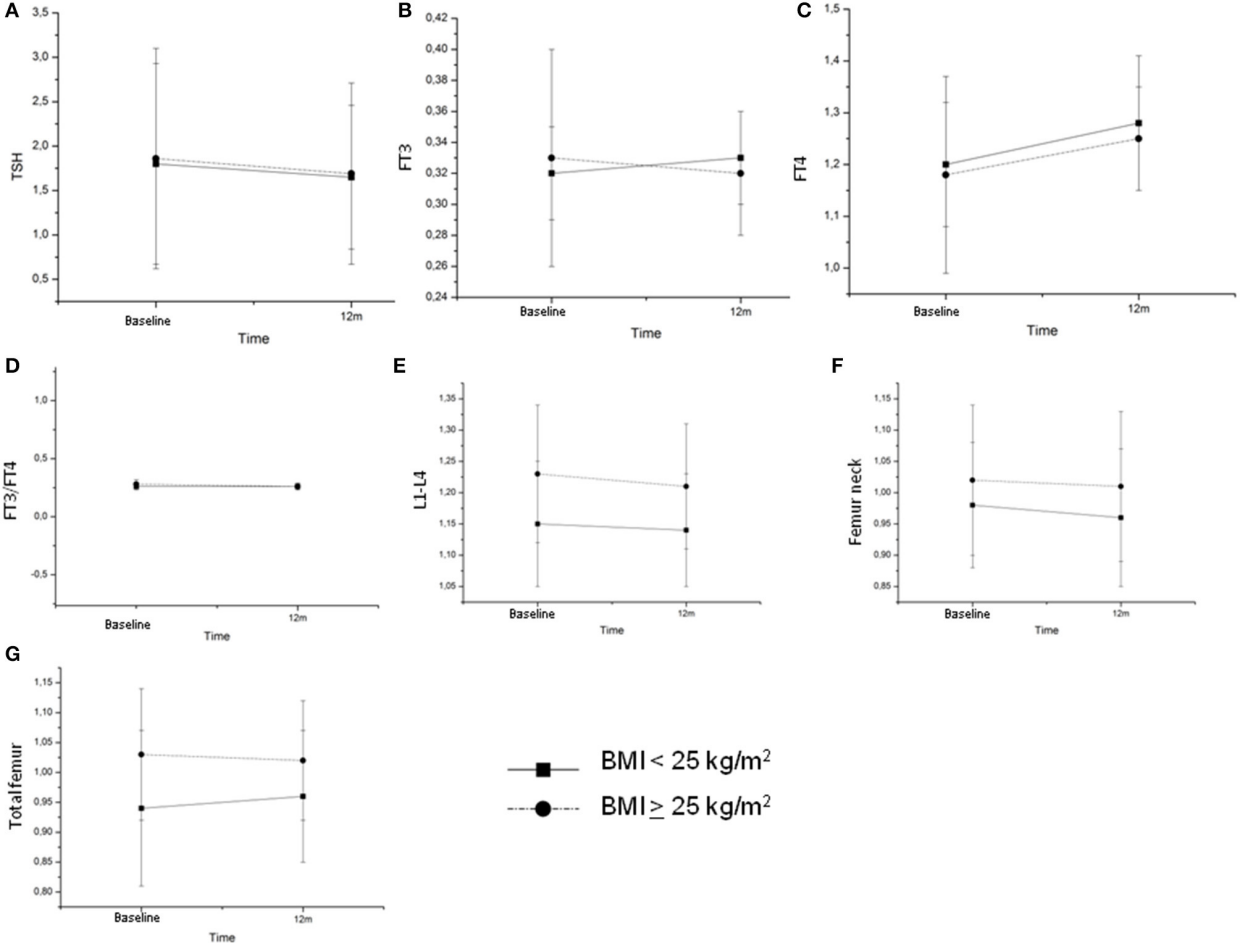
Comparison of thyroid profile and bone mineral density between baseline and 12 months in women of normal weight and overweight. Time comparisons of TSH **(A)** and FT3 concentrations **(B)** did not show differences. **(C)** Time comparison, *p* < 0.0001. **(D)** Comparison between groups at baseline: overweight > normal weight, *p* = 0.0372. **(E)** Comparison between groups at baseline: overweight > normal weight, *p* = 0.0177. **(F)** The signal of *p*-value: *p* = 0.0394. **(G)** Comparison between groups at baseline: overweight > normal weight, *p* = 0.0115. No other differences.

Evaluation of the thyroid hormonal profile, considering the follow-up time and the BMI groups, did not show differences regarding TSH and free T3 concentrations. However, both groups showed a significant increase in mean concentration of free T4 at 12 months (*p* < 0.0001) (Figure 1).

Relating to calcium metabolism and vitamin D, for both groups, there was an increase in calcium (*p* < 0.0001), ALP (*p* < 0.0001), and albumin (*p* = 0.0015) at 12 months. After analysis of the group–time interaction, vitamin D concentrations were significantly lower at 12 months in the group of normal weight women (*p* = 0.0132), with no differences for the overweight group (Table 1).

Among BMD parameters, we verified that both groups increased femur neck BMD at 12 months by time effect (*p* = 0.0394) (Figure 1).

Both groups significantly increased the total mass at 12 months (*p* = 0.0196), and there were no differences in concentrations of leptin and adiponectin between groups (Table 2).

### Associated Factors—Multiple Linear Regression Analysis

Increased BMD of the femoral neck was associated with moderate coffee intake. There were no other associations with bone mass, and no associations were found with the thyroid hormone profile (Table 3).

**Table 3:** Multiple linear regression analysis of the variables associated with BMD and corporal composition variations between 12 months and baseline.

**Associated variables**	**Coefficient (SE)**	***p* value**
**BMD femur neck**		
Moderate coffee intake	11.77778	0.0233
**Total mass (kg)**		
Normal BMI	−8.14838	0.0134
ALP	0.32977	0.0098
Leptin	6.52114	<0.0001
**Fat mass**		
Normal BMI	−10.69886	0.0021
Physical exercises practice	−10.61256	0.0073
Leptin	5.75885	0.0004
PTH	0.38107	0.0135
**% Fat mass**		
Normal BMI	−11.30556	0.0049

*BMD, bone mineral density; ALP, alkaline phosphatase; PTH, intact parathyroid hormone; SE, standard error of the mean. Predictive variables considered in the model: Group (DMPA/TCu380A IUD); variables of Body Composition, BMD, thyroid hormones, and bone metabolism; smoking habits; coffee intake; physical exercises practice, leptin, adiponectin*.

Leptin and ALP were associated with total mass, while leptin and PTH were associated with fat mass. The normal BMI was inversely associated with the total mass, mass, and percentage of fat mass, and regular physical activity was inversely associated with fat mass (Table 3).

## Discussion

This study of euthyroid women with no known diseases showed no association between the thyroid hormone profile within the euthyroid range and BMD or body composition variations. The only factor associated with BMD variation was moderate coffee intake. Concerning body composition, associations were found between factors that are known to be related. There was a direct association of total and fat mass with leptin and an inverse association between normal BMI and regular physical activity.

The division of the sample into comparison groups by BMI—women of adequate weight and overweight—was done to gather possible interferences between excess body weight and the metabolism of fat, calcium, and thyroid hormones, already well-established in obese individuals (22–25). In any case, it is unclear whether thyroid hormones would be associated with changes in fat and calcium metabolism in non-obese individuals. In our study, the overweight group showed expected differences in the evaluated body composition parameters, such as a greater total and fat mass, higher BMD in the lumbar spine and total femur, and greater peripheral conversion of T4 in T3, reinforcing the impact of body weight on these variables.

Although there were no differences between groups with regard to the thyroid hormonal profile in the baseline analysis, it was observed that the T3/T4 ratio was higher in the overweight group. This is a surrogate measure of deiodinase activity indicative of higher peripheral conversion from T4 to T3, and could mean that the thyroid response was adequate for the needs of these overweight women. A study suggested that increased body weight or adiposity could generate higher levels of FT3 in subjects undergoing levothyroxine replacement (23). In addition, an association between body weight and increased FT3 has been observed in euthyroid individuals (24). These authors referred that T3 concentration was correlated with nutrition status, and that moderate weight loss resulted in a decrease of only T3, suggesting a decrease in the peripheral conversion from T4 to T3 (24). However, some studies have suggested that variations in FT3 levels, the active thyroid hormone, are a consequence of, rather than a direct contributor to, body weight (23). Moreover, other authors found that modest increases in serum TSH concentrations within the reference range may be associated with weight gain (15, 26).

On the other hand, the time interval for reassessment could reveal changes between groups within the parameters studied. Thus, at 12 months, an increase in FT4, calcium, and ALP concentrations were observed in normal BMI and overweight groups. The action of time in both groups showed an increase in body mass and BMD of the femoral neck, probably as a consequence of increased body weight. At 12 months, we observed increases in calcium and ALP concentrations. An increase in ALP was also associated with total mass. These results could be due to an increase in bone metabolism, to the intake of food rich in calcium and to physical exercise. Although a concomitant increase in FT4 had been observed, there was no relationship between the thyroid hormonal profile and changes in BMD or body composition.

As expected, at baseline, the overweight group showed a higher BMD in the lumbar spine and total femur, which correlate with previous studies that showed an association between BMI and BMD (27, 28). We observed a higher concentration of leptin in the overweight group in the baseline analysis. Studies have shown that although leptin is an anorexigenic hormone, in obese individuals, the circulating level is increased due to leptin resistance (29). The same group, as expected, showed higher total mass, fat mass, and percentage of fat mass in relation to the normal weight group.

Vitamin D concentrations were similar in both groups at baseline; however, they decreased in the normal weight group at 12 months. A possible explanation for this result could be the significant weight gain observed at 12 months, which could have contributed to an increase in the vitamin D reservoir available in the fat tissue and, consequently, a decrease in serum vitamin D concentrations. A study that evaluated vitamin D in the different deposits of adipose tissue of normal and obese women found that the enlarged adipose mass in obese individuals serves as a reservoir for vitamin D and that the increased amount of vitamin D required to saturate this depot may predispose obese individuals to inadequate serum 25OH vitamin D (30). At the same time, as the serum level of vitamin D decreased in this group, possibly by weight gain, we found that PTH was associated with fat mass, showing the interaction between both to maintain calcium homeostasis.

The higher femoral neck BMD was associated with moderate coffee intake. Studies that have described the phytosteroid and antioxidant actions of substances in coffee that could act on the bone (31) have found that coffee has an adverse effect on BMD (32). However, in Brazil, the preparation of coffee is concentrated and the habit of consuming more and several times a day is common (33).

Among the limitations of this study are the small number of women and the 12 month interval for comparison of the variables. It is possible that a longer follow-up time and a larger sample number could contribute to greater certainty in the results. In addition, this study was a secondary analysis of data from women using two different contraceptive methods—DMPA and an IUD with copper. DMPA has been known to contribute to decreased BMD among users (34), and although we have adjusted all the statistical analyses for the use of the method, it is still possible that this may have influenced the results. On the other hand, the strength of this study was to evaluate the same cohort of women—eutrophic, euthyroid, and without other diseases or history of known disease—in a period of 1 year while monitoring their lifestyle habits. To our knowledge, this is the first study of women with these characteristics.

Unlike in individuals with thyroid dysfunctions, in non-obese euthyroid women, the thyroid hormone profile was not associated with variations in BMD and body composition at a 1 year follow-up.

## Data Availability Statement

The datasets generated for this study are available on request to the corresponding author.

## Ethics Statement

The studies involving human participants were reviewed and approved by ClinicalTrials.gov number NCT01527526. Project approved by the ethics committee of the Faculty of Medical Sciences of University of Campinas. The patients/participants provided their written informed consent to participate in this study.

## Author Contributions

AQ-M, DZ-W, PS, VM-K, and AF wrote the research project and were responsible for collecting the information and writing the draft of this article. DZ-W, LB, and AF were responsible for interpreting the information and reviewing and approving the final version of the manuscript.

### Conflict of Interest

The authors declare that the research was conducted in the absence of any commercial or financial relationships that could be construed as a potential conflict of interest.
